# Monitoring of Levosimendan Administration in Patients with Pulmonary Hypertension Undergoing Cardiac Surgery and Effect of Two Different Dosing Schemes on Hemodynamic and Echocardiographic Parameters

**DOI:** 10.3390/ph16060815

**Published:** 2023-05-30

**Authors:** Panagiotis Ftikos, Areti Falara, Panagiota Rellia, Evangelos Leontiadis, George Samanidis, Natalia Kamperi, Artemios Piperakis, Constantin Tamvakopoulos, Theofani Antoniou, Kassiani Theodoraki

**Affiliations:** 1 Department of Anesthesiology, Onassis Cardiac Surgery Center, 176 74 Athens, Greece; pftikos@yahoo.gr (P.F.); areti_f@hotmail.com (A.F.); grellia@yahoo.gr (P.R.); antoniou_fani@yahoo.gr (T.A.); 2 Department of Cardiology, Onassis Cardiac Surgery Center, 176 74 Athens, Greece; evanleont@gmail.com; 3 Department of Cardiac Surgery, Onassis Cardiac Surgery Center, 176 74 Athens, Greece; gsamanidis@yahoo.gr; 4 Center of Clinical Research, Experimental Surgery and Translational Research, Division of Pharmacology-Pharmacotechnology, Biomedical Research Foundation, Academy of Athens, 115 27 Athens, Greecectamvakop@bioacademy.gr (C.T.); 5 Department of Anesthesiology, Aretaieion University Hospital, National and Kapodistrian University of Athens, 115 28 Athens, Greece

**Keywords:** levosimendan, pulmonary hypertension, pharmacokinetics, mass spectrometry

## Abstract

Introduction: The perioperative management of patients with pulmonary hypertension (PH) undergoing cardiac surgery represents one of the most challenging clinical scenarios. This fact mainly depends on the relationship existing between PH and right ventricular failure (RVF). Levosimendan (LS) is an inodilator that might be an effective agent in the treatment of PH and RVF. The aim of this study was to examine the impact of the duration of cardiopulmonary bypass (CPB) on the therapeutic drug monitoring of LS and to evaluate the effect of preemptive administration of LS on perioperative hemodynamic and echocardiographic parameters in cardiac surgical patients with preexisting PH. Materials and Methods: In this study, LS was administered in adult patients undergoing cardiac surgery before CPB in order to prevent exacerbation of preexisting PH and subsequent right ventricular dysfunction. Thirty cardiac surgical patients with preoperatively confirmed PH were randomized to receive either 6 μg/kg or 12 μg/kg of LS after the induction of anesthesia. The plasma concentration of LS was measured after CPB. In this study, a low sample volume was used combined with a simple sample preparation protocol. The plasma sample was extracted by protein precipitation and evaporated; then, the analyte was reconstituted and detected using specific and sensitive bioanalytical liquid chromatography with mass spectrometry (LC-MS/MS) methodology. The clinical, hemodynamic, and echocardiographic parameters were registered and evaluated before and after the administration of the drug. Results: A fast bioanalytical LC-MS/MS methodology (a run time of 5.5 min) was developed for the simultaneous determination of LS and OR-1896, its main metabolite in human plasma. The LC-MS/MS method was linear over a range of 0.1–50 ng/mL for LS and 1–50 ng/mL for its metabolite OR-1896. Measured plasma concentrations of LS were inversely related to the duration of CPB. LS administration before CPB during cardiac surgery was effective in reducing pulmonary artery pressure and improving hemodynamic parameters after CPB, with a more pronounced and durable effect of the drug at the dose of 12 μg/kg. Additionally, administration of LS at a dose of 12 μg/kg in cardiac surgical patients with PH before CPB improved right ventricular function. Conclusion: LS administration decreases pulmonary artery pressure and may improve right ventricular function in patients with PH undergoing cardiac surgery.

## 1. Introduction

Pulmonary hypertension (PH) constitutes a significant challenge in the perioperative care of patients presenting for cardiac surgery [1,2]. This fact mainly depends on the relation between PH and right ventricular failure (RVF), which carries a significant burden of morbidity and mortality following cardiac surgery, necessitating early recognition and prompt treatment [3,4,5].

RVF in the perioperative setting is associated with mortality rates ranging up to 22–90%, as well as significant complications, including increased need for inotropic and mechanical circulatory support, prolonged mechanical ventilation, and prolonged length of intensive care unit (ICU) and in-hospital stay [6,7].

The most frequent type of PH in patients presenting for cardiac surgery is PH secondary to left heart disease [8,9]. Mitral valve disease, and in particular mitral stenosis, is strongly associated with PH [10]. PH also frequently occurs in patients with left ventricular systolic and diastolic dysfunction [11]. However, as the average age and the comorbidities of cardiac surgical patients have increased in the last years, any form of PH is likely to be confronted in this group of patients. Moreover, with the aging population and the associated increased severity of illness, the prevalence of PH in patients presenting for cardiac surgery is rising [12].

Regardless of the underlying cause, PH may be exacerbated during cardiac surgery due to several causes. The most important causes involved include systemic inflammatory response and pulmonary reperfusion syndrome after cardiopulmonary bypass (CPB) [13,14,15], administration of protamine [16,17], transfusion of blood products [18,19], low cardiac output syndrome (LCOS) and patient prosthesis mismatch after heart valve surgery [20,21].

Exacerbation of preexisting PH during cardiac surgery is related to increased right ventricular (RV) afterload and may lead to RVF. The most important consequence of uncontrolled PH is RVF, which in turn is accompanied by decreased pulmonary blood flow, decreased left ventricular (LV) preload, and decreased cardiac output (CO) and arterial blood pressure (BP). The subsequent drop in coronary blood flow further worsens RV function, and consequently, a vicious cycle is established that eventually leads to supra-systemic RV pressures and cardiovascular collapse [4,5,6].

RVF in the perioperative setting is difficult to diagnose and even more demanding to treat. It typically presents intraoperatively as difficulty in separation from CPB and as LCOS or multi-organ dysfunction in the postoperative period. Currently, there are no established evidence-based guidelines for the perioperative management of RVF in cardiac surgical patients with PH. The principal management goal is to prevent RVF by reducing RV afterload, optimizing preload, and enhancing RV contractility [22,23,24].

In this context, several pharmacologic agents administered intravenously or via inhalation have been used in order to prevent the worsening of PH during cardiac surgery and treat RVF. These agents include prostaglandins, nitric oxide (NO), milrinone, adrenergic agents, and levosimendan (LS) [25,26,27]. LS is a calcium-sensitizing agent with inotropic, vasodilatory, and cardioprotective properties [28]. LS enhances myocardial contractility by increasing the affinity of myocardial troponin C to calcium. In contrast to other inotropic agents, the positive inotropic action of LS does not occur at the expense of calcium overload or increased myocardial oxygen demand [29]. Additionally, LS displays vasodilatory effects by the opening of adenosine triphosphate (ATP)-dependent K^+^ channels in vascular smooth muscle cells, causing vasodilation in both arterial and venous smooth muscle cells. The cardioprotective properties of LS are associated with the opening of mitochondrial ATP-dependent K^+^ channels in the cardiomyocytes, providing protection against ischemia-reperfusion injury, apoptosis, and oxidative stress [30]. LS has a fast onset of action, a short half-life of one hour, and a prolonged effect mainly due to the formation of an active metabolite, OR-1896 [31].

Given the vasodilatory properties of LS, it could distend pulmonary vasculature and vasculature of the venous system, causing a reduction in both RV preload and afterload, while, given the inotropic action of the drug, it could enhance RV contractility [32,33]. Although LS is a potentially favorable agent in treating PH and associated RVF, few data exist regarding its use in patients with PH undergoing cardiac surgery and the impact of extracorporeal circulation on its pharmacokinetic and pharmacodynamic properties.

The aim of the study is to examine the pharmacokinetic and pharmacodynamic properties of LS in patients with PH undergoing cardiac surgery with the use of CPB, evaluate the efficacy of its administration prior to CPB in preventing exacerbation of PH and RVF, and identify the most effective dose of the drug in the perioperative context.

## 2. Results

In total, 34 patients were screened for enrollment in this study. Of those, 30 patients fulfilled the inclusion criteria and completed the study. Therefore, the sample consisted of 30 patients (56.7% females) with a mean age of 68.9 years (SD 11.0 years). Half of the patients (N = 15) received a dose of 6 μg/kg, and the other half (N = 15) received a dose of 12 μg/kg. Patients’ characteristics were similar in both dose groups (Table 1). No in-hospital mortality was recorded.

The median LS blood plasma concentration at 20 min after CPB was 6.7 ng/mL. Throughout the follow-up period, LS plasma concentration values were not significantly different between the two study groups at each time point. LS blood concentration values diminished significantly over the follow-up period in both dose groups, with a similar degree of reduction (Table 2). A significant and negative association was found between the CPB duration and LS plasma concentration change over the follow-up period (*p* = 0.007). Significantly higher values of LS at 20 min and 6 h after CPB were found when CPB duration was shorter compared to when CPB duration was longer (Supplementary Table S1).

At baseline, hemodynamic parameters were not significantly different between the two dose groups (Table 3). At 20 min after CPB, CO, CI, SV, and SVI were significantly greater in patients who received a dose of 12 μg/kg. Similarly, at the end of the surgery, CO and SV were significantly greater in patients who received a dose of 12 μg/kg as compared to those who received 6 μg/kg. Moreover, SV at 2 h after ICU admission was significantly greater in patients who received a dose of 12 μg/kg, while SPAP was significantly lower in the same group of patients at the same time point. MAP, DPAP, PCWP, SVR, SVRI, PVR, and PVRI were reduced significantly over the follow-up period in both dose groups to a similar degree. CO and CI increased significantly over the follow-up period in both dose groups to a similar degree.

**Figure 1:**
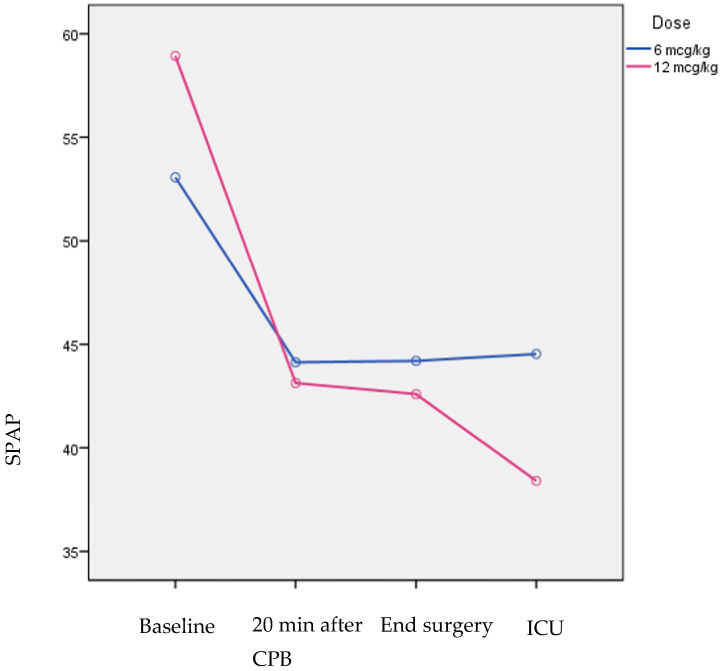
SPAP changes across the follow-up period by dose group.

SPAP (Figure 1), MPAP, and MPAP/MAP decreased significantly in both dose groups over the follow-up period, but the decrease was significantly greater in patients who received a dose of 12 μg/kg. LVEF and TAPSE values were not significantly different between the two dose groups across the follow-up period (Table 4). LVEF did not change significantly over time in either group. The degree of TAPSE change differed significantly over the follow-up period. More specifically, TAPSE increased significantly only in the group of 12 μg/kg, while in the group of 6 μg/kg, it did not change significantly.

SPAP, DPAP, MPAP, and MPAP/MAP diminished significantly across the follow-up time in patients with plasma levels of LS < 6.7 ng/mL at 20 min after CPB as well as in patients with plasma levels of LS > 6.7 ng/mL at 20 min after CPB (Supplementary Table S2). The degree of change was similar in both patient groups, and no significant differences between the two groups were found at each time point. LVEF values increased across the follow-up period in patients with LS plasma levels > 6.7 ng/mL at 20 min after CPB (*p* = 0.023), but no statistically significant difference regarding the LVEF change over time was found between patients with plasma levels of LS > than 6.7 ng/mL at 20 min after CPB and patients with plasma levels of LS < than 6.7 ng/mL at 20 min after CPB (*p* = 0.101). Similarly, TAPSE values increased over time in patients with LS levels > 6.7 ng/mL at 20 min after CPB (*p* = 0.001). The degree of change of TAPSE differed significantly between the two groups, as indicated by the significant interaction effect of the analysis. Specifically, the increase was significantly greater in patients with LS plasma concentration > 6.7 ng/mL at 20 min after CPB (*p* = 0.015) (Supplementary Table S3). Length of ICU stay and duration of hospitalization was similar in patients with LS blood concentration less than 6.7 ng/mL at 20 min after CPB as compared to patients with LS blood concentration more than 6.7 ng/mL at 20 min after CPB (Supplementary Table S4).

Finally, no statistical difference regarding the need for vasopressor (norepinephrine) and additional inotropic support (dobutamine or epinephrine) was found between the two groups of patients.

## 3. Discussion

According to the results of the present prospective randomized clinical study, LS administered both at a dose of 6 μg/kg or 12 μg/kg after induction of anesthesia and before CPB in cardiac surgical patients with preoperatively confirmed PH is effective in reducing SPAP values after CPB in comparison to baseline values. Although both doses are effective in reducing SPAP, the decrease is greater when LS is administered at a dose of 12 μg/kg. In addition, both doses are effective in increasing CO and CI, but only the administration of LS at a dose of 12 μg/kg is related to enhanced RV function, as shown by increased TAPSE values. No significant difference in the incidence of hypotension and need for vasopressor support was found between the two groups of patients receiving LS at a dose of 6 μg/kg or 12 μg/kg.

These findings suggest that the prophylactic use of LS in patients with PH undergoing cardiac surgery might inhibit or reduce the degree of exacerbation of preexisting PH and potentially subsequent RVF after CPB. This fact could be attributed to the vasodilatory and inotropic properties of LS as well as to the anti-inflammatory action of the drug, given that the exacerbation of preexisting PH during cardiac surgery is strongly associated with the systemic inflammatory response induced by extracorporeal circulation.

Regarding the pharmacokinetic profile of LS during cardiac surgery, the results of our study indicate that plasma levels of the drug are in inverse proportion with the duration of CPB. OR-1896 was not present in a detectable concentration in the clinical samples analyzed at 20 min, 6, 12, 24, and 80 h after CPB. This finding could indicate that CPB and extracorporeal circulation could severely affect the pharmacokinetic profile of LS. In clinical practice, this fact could translate into a need for adjustment of the dose of LS administered before CPB in patients undergoing cardiac surgery to obtain therapeutical plasma levels of the drug and, thus, clinical effectiveness. The relation between CPB and the pharmacokinetics of LS could represent a potential reason for subtherapeutic plasma levels of LS and altered metabolism of the drug and its active metabolite, OR-1896, after CPB. Consequently, the inverse relationship between the length of CPB and extracorporeal circulation and the pharmacokinetic profile of LS could create confusion concerning the effectiveness of the drug in cardiac surgery and, by extension, be a potential factor explaining the heterogeneous results of clinical trials with LS in the context of cardiac surgery [34,35].

Plasma concentrations associated with LS efficacy were assessed in an open-label, non-randomized phase II study in patients diagnosed with heart failure (New York Heart Association III-IV), whereby a 24-h continuous infusion of LS at a dose of 0.2 mcg/kg/min produced peak plasma concentrations of 62.6 ng/mL [36]. According to the results of our study, even low values of plasma concentration of LS obtained after a single bolus dose of 6 μg/kg or 12 μg/kg of the drug are efficient in reducing SPAP, while improvement of RV contractility as shown by TAPSE values is observed only with plasma levels of LS higher than 6.7 ng/mL. Of note, the drug’s vasodilatory effects on systemic vasculature are observed, according to our findings, at both low and high doses of LS.

LS has been in clinical use for over two decades and has been the subject of considerable evaluation in a vast range of clinical applications. The main indication of LS is the management of acutely decompensated chronic heart failure, and most of the evidence concerns the effect of the drug on the left ventricular performance. Beyond its main indication, LS has been evaluated with conflicting results in various perioperative settings, including coronary artery bypass graft surgery (CABG) and valve surgery in patients with low ejection fraction [37,38,39], heart transplantation [40] and weaning from venoarterial extracorporeal membrane oxygenation (V-A ECMO) [41]. Evidence for LS treatment in PH and RVF is currently limited, and it is not possible to extrapolate knowledge from studies with LS on the left ventricular function to right ventricular performance.

Although the present literature is not extensive, the existing data suggest that LS potentially has a beneficial role in treating PH and associated RVF resulting from various etiologies, including pulmonary arterial hypertension, left heart disease, and congenital heart disease [32]. However, limited data exist concerning the intraoperative use of LS in cardiac surgical patients with PH, and there are even less available data regarding the relation between extracorporeal circulation and the pharmacokinetic and pharmacodynamic properties of the drug. Ebade et al. demonstrated that LS was superior to dobutamine in improving CI and lowering MPAP in children younger than 4 years with PH due to congenital heart disease undergoing cardiac surgery [42], while Abdelbaser et al. suggested that both intravenous and inhaled LS were effective in reducing pulmonary artery pressure in pediatric cardiac surgical patients with PH [43]. It is very interesting that apart from the intravenous use of LS, some studies support the administration of the drug via inhalation in order to avoid systemic hypotension that could reduce RV perfusion [34,43].

Our findings are in agreement with the existing data and support that LS could have a favorable role in preventing the worsening of PH during cardiac surgery and superimposed RVF. In clinical practice, given the high risk of RVF faced by cardiac surgical patients with preexisting PH, administration of LS prior to CPB, in association if necessary with vasopressors, could be protective against exacerbation of PH and subsequent RVF during weaning and after CPB. According to our findings, LS should be administered at a dose of 12 μg/kg to impact RV contractility positively. Alternatively, LS plasma levels above 6.7 ng/mL should be achieved for this goal.

This study has a few limitations, in addition to the small sample size. Patients with PH due to valvular disease may demonstrate a decrease in pulmonary artery pressure after surgical correction of valvular pathology due to the release of left-sided obstruction, independently of pharmacological treatment. However, in our study, the persistence of a moderate level of pulmonary hypertension in both groups was documented after surgery. The residual pulmonary hypertension was due either to morphologic changes in the pulmonary vasculature and/or increased pulmonary vasculature reactivity due to systemic inflammatory response. Moreover, the study was designed to compare the effectiveness of different doses of the same drug that, according to the existing literature, are favorable in treating PH. Nevertheless, the absence of a control group receiving a placebo or another drug (i.e., milrinone) represents a limitation. An additional limitation is that the echocardiographic assessment of right ventricular function was performed only using TAPSE. Combining more than one echocardiographic method of right ventricular function assessment would allow us to better evaluate right ventricular performance and distinguish normal from abnormal function. Furthermore, the follow-up period was relatively short, including the intraoperative period and the first two hours of the postoperative period in the ICU. It would definitely be interesting to extend the follow-up period of hemodynamic and echocardiographic data over the first 24 h postoperatively. However, the comparison of hemodynamic measurements involving intubated patients in mechanical ventilation with hemodynamic data involving spontaneous ventilating patients would not be possible. Finally, regarding LC-MS/MS methodology, the method was partially validated based on EMA guidelines for the quantification of drugs in biological fluids (bioanalytical method validation). The LC-MS/MS method for the quantification of LS and OR-1896 in human blood plasma was investigated for specificity, linearity, accuracy, and precision. The linearity in the range of 0.1–50 ng/mL for LS and 1–50 ng/mL for OR-1896 was determined over multiple runs with R values typically > 0.999. Specificity was evaluated by monitoring the presence of LS, OR-1896, and IS in blank pooled plasma. No interfering peaks were detected in blank samples. Matrix effect, recovery, and stability studies were beyond the scope of this pilot clinical protocol and will be further investigated in future studies.

## 4. Materials and Methods

### 4.1. Study Population

This prospective, randomized, single-center, and interventional study was approved by the Hospital’s (Onassis Cardiac Surgery Center) Ethics Committee (N^0^ 676 OF 006/B’ CYCLE -23/3/20), and informed consent was obtained from each patient before the operation. The study was prospectively registered on clinicaltrials.org before patient enrollment under the number NCT04599816.

The study population consisted of 30 patients with severe PH due to left heart disease undergoing elective cardiac surgery. Patients were considered to have severe PH if pulmonary artery systolic pressure (SPAP) was greater than 55 mmHg or mean pulmonary arterial pressure (MPAP) was greater than 25 mmHg, as estimated by preoperative transthoracic or transesophageal echocardiography (TEE) or right heart catheterization. The inclusion criteria were adult patients with PH undergoing elective cardiac surgery with the use of extracorporeal circulation. The exclusion criteria were left ventricular ejection fraction (LVEF) < 30%, severe renal failure and hepatic failure, acute or chronic thromboembolic disease, and chronic obstructive pulmonary disease (COPD).

### 4.2. Randomization of Patients

Patients were randomly assigned into two treatment groups: Group A, receiving LS at a dose of 6 μg/kg, and Group B, receiving LS at a dose of 12 μg/kg. Randomization was performed using a computer-generated random code. The allocation code was concealed in an envelope that was opened by the nurses participating in the study who were also in charge of preparing the medication.

### 4.3. Intraoperative Anesthetic Management

Premedication, monitoring, anesthesia, and mechanical ventilation were standardized. Apart from standard monitoring for cardiac anesthesia, a pulmonary artery catheter (Swan-Ganz) and TEE were used in all patients. The presence of PH was confirmed by the Swan-Ganz catheter after anesthetic induction, and a comprehensive TEE exam was performed in all patients.

Anesthetic induction in all patients was performed with intravenous doses of midazolam 0.05 mg/kg, fentanyl 2 μg/kg, and rocuronium bromide 1 mg/kg. For maintenance, all patients received sevoflurane at an end-tidal concentration of 0.5% to 2% and intravenous maintenance doses of midazolam and fentanyl every hour, aiming for bispectral index (BIS) values of 40 to 50. Protective ventilation (VT: 6 mL/kg, PEEP: 5) was used in all patients maintaining normocapnia (PCO_2_ 35–40 mmHg)—blood transfusion practice aimed at maintaining hemoglobin concentration between 9 and 10 g/dL.

### 4.4. Intraoperative Hemodynamic Parameters

All perioperative hemodynamics parameters and TTE findings were evaluated by a Swan-Ganz catheter (7.5F, Edwards Lifescience, Irvine, CA, USA) and a Vivid 3 echocardiography device (General Electric, Hamburg, Germany). Variables measured or calculated included heart rate (HR), systolic arterial pressure (SAP), diastolic arterial pressure (DAP), mean arterial pressure (MAP), SPAP, diastolic pulmonary artery pressure (DPAP), MPAP, mean pulmonary to mean systemic pressure ratio (MPAP/MAP), central venous pressure (CVP), pulmonary capillary wedge pressure (PCWP), stroke volume (SV), CO and cardiac index (CI), systemic vascular resistance (SVR), systemic vascular resistance index (SVRI), pulmonary vascular resistance (PVR), pulmonary vascular resistance index (PVRI), tricuspid annular plane systolic excursion (TAPSE) and LVEF. CO was assessed using the thermodilution technique with three injections of room temperature 5% dextrose 10 mL, and PCWP was measured at end-expiration. CO, SV, SVR, and PVR were indexed to body surface area BSA, calculated using the Du Bois formula (Body Surface Area (m^2^) = 0.007184 × Height(cm)^0.725^× Weight(kg)^0.425^.

Hemodynamics were treated according to the following protocol during and after surgery: (1) CVP or PCWP to keep values between 10 to 12 mmHg and 16 to 20 mmHg, respectively, with fluid administration, (2) MAP of 60 to 90 mmHg with norepinephrine 0.05 μg/kg/min increased incrementally by 0.02 μg/kg/min until the MAP was 60 mmHg, (3) if CI < 2 L/min/m^2^ inotropic support was started initially with dobutamine 2–10 μg/kg/min followed (if necessary) by the addition of epinephrine, 0.01–0.1 μg/kg/min.

### 4.5. Transesophageal Echocardiographic Parameters

Intraoperative echocardiographic evaluation was performed by two anesthesiologists and reviewed by an experienced cardiologist. TAPSE was measured in the four-chamber view as the distance between the end-diastolic and end-systolic position of the outer port of the tricuspid annulus. LVEF was calculated using the modified Simson method [44].

### 4.6. ICU Management and ICU Discharge Criteria

Patients were weaned from mechanical ventilation when rewarmed and hemodynamically stable and within normal arterial blood gas values. Patients were discharged from the ICU when the following criteria were met: SpO_2_ > 90% at FiO_2_ of 0.5 by facemask, stable hemodynamics, chest tube drainage <50 mL/h, urine output >0.5 mL/kg/h, and no intravenous inotropic or vasopressor therapy.

### 4.7. Levosimendan Administration and Blood Plasma Concentration Measurement

After induction of anesthesia and confirmation of PH presence, LS diluted in 100 mL (Dextrose 5% Water) was administered with a continuous infusion for 20 min. LS administration was discontinued in the event of an anaphylactic reaction, refractory hypotension (defined as a MAP <60 mmHg despite optimal therapeutic management), or intractable arrhythmias, and the patient was excluded from the protocol and further analysis.

Before and after the administration of LS, the already determined intraoperative hemodynamic and transesophageal echocardiographic parameters were evaluated by monitoring with the Swan-Ganz catheter and TEE and recorded in our database. Hemodynamic measurements and transesophageal echocardiographic evaluation were performed after induction of anesthesia, 20 min after discontinuation of CPB, at the end of the surgery, and two hours after ICU admission.

Blood plasma concentrations of LS were measured at 20 min after discontinuation of CPB and correlated with hemodynamic and TEE parameters evaluated at this point in time. Moreover, the blood plasma concentration of LS was measured at 6, 12, 24, and 80 h after CPB weaning. At the same time points, the blood plasma concentration of OR-1896 was also measured. Blood plasma concentration of LS and OR-1896 was measured using a bioanalytical methodology of liquid chromatography coupled with mass spectrometry (LC-MS/MS). Blood samples (3 mL) for the determination of plasma concentrations of LS and its metabolite OR-1896 were centrifuged within 10 min of sampling. The plasma was separated and transferred into two polypropylene tubes, frozen immediately, and kept at −70 °C until analysis.

### 4.8. LC-MS/MS Methodology

#### 4.8.1. Chemicals

LS (99.9%, LC-MS) and OR-1896 (98.9%, LC-MS) were purchased from MedChemtronica AB (Sollentuna, Sweden). Warfarin used as an internal standard (IS) was obtained from Riedel-de Häen (Seelze, Germany). Human plasma pooled gender was purchased by Sera Laboratories International Ltd., trading as BioIVT (West Sussex, UK). Ammonium acetate (LC-MS grade), formic acid (FA, 99.0%, LC-MS grade), acetonitrile (ACN, LC-MS grade), methanol (MeOH, LC-MS grade), water (LC-MS grade) and dimethylsulfoxide (DMSO, ≥99.7%) were purchased from Fisher Scientific (Fisher Scientific, Loughborough, United Kingdom).

#### 4.8.2. Instrumentation and LC-MS/MS Conditions

For the quantification of LS in human blood plasma, an LC-MS/MS bioanalytical methodology was developed. Chromatographic separation of LS, OR-1896, and warfarin was carried out using a Synergi Fusion-Reversed Phase column (2.0 mm × 50 mm, 4.0 μm) (Phenomenex, CA, USA) with an injection volume of 10 μL at a flow rate of 0.30 mL/min. The optimal LC conditions were as follows: mobile phase A: 10% ACN, 90% water, 2 mM ammonium acetate, 0.1% FA, and mobile phase B: 90% ACN, 10% water, 2 mM ammonium acetate, 0.1% FA. The analyte of interest could be achieved following a gradient elution program within a chromatographic time at 5.60 min: starting from 0% phase B (0.00–0.50 min) to 60% phase B (0.50–1.00 min), 60% phase B (1.00–3.60 min) and from 60% to 0% phase B (3.60–5.60). A SCIEX QTRAP 5500+ (SCIEX, Concord, ON, Canada) was operated in negative ionization mode using the multiple reaction mode (MRM) with a dwell time of 50 msec per transition. High-performance liquid chromatography (HPLC) was performed using an Exion LC AB Sciex, a temperature-controlled column compartment, and an autosampler coupled with a SCIEX QTRAP 5500+ mass spectrometer. The electrospray ionization source conditions were set as follows: electrospray voltage of −4500 V for negative mode, source temperature of 550 °C, curtain gas of 20, ion source gas 1 and gas 2 of 50 psi and 45 psi, respectively. For LS, the transitions *m/z* 279 → 227 and *m/z* 279 → 64 were monitored. For OR-1896, the transitions *m/z* 244 → 201 and *m/z* 244 → 159 were monitored. For the IS, the transition *m/z* 307 → 161 was monitored. An indicative MRM chromatogram depicting LS, OR-1896, and IS in concentrations of 0.5 ng/mL, 5 ng/mL, and 1 ng/mL, respectively, is provided in Supplementary Figure S1.

#### 4.8.3. Preparation of Stock Solutions, Calibration Standard Solutions, and Quality Control (QC) Solutions

Individual superstocks of LS and OR-1896 were prepared in a concentration of 2 mg/mL and 1 mg/mL, respectively, dissolved in DMSO. A stock of 1 mg/mL of IS in MeOH. From these superstocks, subsequent stock solutions for LS (5, 50, 500, 10,000, 100,000 ng/mL), OR-1896 (50, 500, 10,000, 100,000 ng/mL) and IS (1 ng/mL) were prepared in 1:1 (*v*/*v*) ACN/water. Then, mixed calibration standard solutions of LS and OR-1896 were prepared using 1:1 (*v*/*v*) ACN/water as diluent: LS (0.1, 0.25, 0.5, 1, 2.5, 5, 10, 25, and 50 ng/mL) and OR-1896 (1, 2.5, 5, 8, 10, 12.5, 25, 37.5 and 50 ng/mL). A separate set of superstock solutions of LS and OR-1896 were used to prepare QC stock solutions at (8, 800, and 80,000 ng/mL) for LS and (50, 500, 5000, and 100,000 ng/mL) for OR-1896. Then, mixed QC solutions were prepared using 1:1 (*v*/*v*) ACN/water as diluent: LS (0.1, 0.2, 20, and 40 ng/mL) and OR-1896 (1, 2, 20, and 40 ng/mL).

#### 4.8.4. Plasma Samples

Calibration curves of LS (0.1–50 ng/mL) and OR-1896 (1–50 ng/mL) in plasma and QC samples were prepared by spiking 50 μL of plasma with 50 μL of calibration standard solutions or QC solutions of analytes followed by 50 μL of IS. The extraction of analytes from human plasma was performed by protein precipitation using 400 μL cold acetonitrile. Samples were then vortexed and centrifuged for 10 min at 13,000 rpm. The supernatant was collected and evaporated using a Centrivap vacuum concentrator of Labconco (Kansas City, MO, USA). Dried samples were resuspended in 400 μL mobile phase A and vortexed. Then, samples were transferred into a 96-well plate for LC-MS/MS analysis using 10 μL as injection volume. All plasma samples received from patients were thawed at room temperature for the analysis. At first, samples were screened for the presence of the IS, as Warfarin is a commonly used anticoagulant agent. When samples were screened as free of IS, they were extracted and analyzed as described above. The LC-MS/MS method for the quantification of LS and OR-1896 in human blood plasma was investigated for specificity, linearity, accuracy, and precision. Data for accuracy and precision of the developed LC-MS method for the quantification of LS and OR-1896 in human plasma are provided in Appendix A (Table A1 and Table A2).

### 4.9. Power Analysis

The power analysis was conducted for a single, two-group between-subjects factor and a single within-subjects factor assessed over four time points. For this design, 30 participants (15/study group) achieves a power of 0.85 for the between-subjects main effect at an effect size of 0.45, a power of 0.90 for the within-subjects main effect at an effect size of 0.25, and a power of 0.90 for the interaction effect at an effect size of 0.25.

### 4.10. Statistical Analysis

Quantitative variables were expressed as mean (standard deviation) or median (interquartile range). Qualitative variables were expressed as absolute and relative frequencies. Student’s *t*-tests and Mann–Whitney tests were used for the comparison of continuous variables between the two groups. For the comparison of proportions, the chi-square and Fisher’s exact tests were used. Repeated measurements analysis of variance (ANOVA) was used to evaluate the changes observed in all patients’ indexes among the different groups over the follow-up period. All reported *p* values are two-tailed. Statistical significance was set at *p* < 0.05, and analyses were conducted using SPSS statistical software (version 22.0).

## 5. Conclusions

In conclusion, according to the results of the present randomized study performed on a small group of patients, the prophylactic administration of LS at a single bolus dose of 12 μg/kg before CPB was effective in reducing pulmonary artery pressure and improving right ventricular performance in patients with preexisting PH undergoing cardiac surgery. Moreover, this study demonstrated that the plasma concentration of LS administered prior to CPB is inversely related to the duration of CPB. More research is needed in order to elucidate the relationship between extracorporeal circulation used in cardiac surgery and the pharmacokinetics of LS and extrapolate knowledge concerning the appropriate dose of the drug that should be used in the context of cardiac surgery. Undoubtedly, larger and well-designed studies are necessary to validate LS’s clinical efficacy and pharmaceutical role in the perioperative anesthetic management of this important group of cardiac surgical patients.

## Figures and Tables

**Table 1:** Sample characteristics by group.

	Dose		P
	6 μg/kg (N = 15; 50%)	12 μg/kg (N = 15; 50%)	
	N (%)	N (%)	
Gender			
Females	9 (60.0)	8 (53.3)	0.713 +
Males	6 (40.0)	7 (46.7)	
Age (years), mean (SD)	69.9 (14.3)	67.9 (6.5)	0.615 ‡
BSA (m^2^), mean (SD)	1.8 (0.2)	1.9 (0.2)	0.338 ‡
Type of surgery			
ASD	0 (0.0)	1 (6.7)	-
AVR	2 (13.3)	0 (0.0)	
AVR-MV repair-TV repair	2 (13.3)	2 (13.3)	
AVR-MVR	2 (13.3)	2 (13.3)	
AVR-MVR-GABG	0 (0.0)	1 (6.7)	
CABG + MV repair	2 (13.3)	2 (13.3)	
MV repair	1 (6.7)	1 (6.7)	
MV repair + TV repair	1 (6.7)	1 (6.7)	
MVR	4 (26.7)	5 (33.3)	
MVR REDO	1 (6.7)	0 (0.0)	
CPB duration (minutes), mean (SD)	137.5 (46.6)	150.5 (63.1)	0.526 ‡
Patients in need of norepinephrine administration	9 (60.0)	9 (60.0)	>0.999 +
Norepinephrine dose (μg/kg/min), mean (SD)	0.1 (0.1)	0.1 (0.1)	0.522 ‡
Patients in need of dobutamine administration	9 (60)	4 (26.7)	0.065 +
Dobutamine dose (μg/kg/min), mean (SD)	3.2 (1.7)	3.8 (1.3)	0.548 ‡
Patients in need of epinephrine administration	1 (7.7)	0 (0.0)	>0.999 ++
Epinephrine dose (μg/kg/min), mean (SD)	0.1 (-)	0 (-)	
ICU length of stay (hours), mean (SD)	103.6 (53.1)	137.3 (274.1)	0.644 ‡
Duration of hospitalization (days), median (IQR)	9 (7–14)	8 (6–10)	0.118 ‡‡

+ Pearson’s x^2^ test; ++ Fisher’s exact test; ‡ Student’s *t*-test; ‡‡ Mann–Whitney test. BSA: body surface area, ASD: atrial septal defect, AVR: aortic valve replacement, MV REPAIR: mitral valve repair, TVP: tricuspid valve annuloplasty, MVR: mitral valve replacement, CABG: coronary artery bypass graft, REDO: repeat surgery, CPB: cardiopulmonary bypass, ICU: intensive care unit.

**Table 2:** LS changes over the follow-up period, by group.

	LS					
	20 min after CPB (ng/mL)	6 h (ng/mL)	12 h (ng/mL)	Change to 12 h		
Dose	Mean (SD)	Mean (SD)	Mean (SD)	Mean (SD)	P^2^	P^3^
6 μg/kg	5.29 (3.72)	1.98 (1.38)	0.18 (0.18)	−5.11 (3.71)	0.001	0.108
12 μg/kg	8.21 (5.56)	3.33 (2.79)	0.2 (0.23)	−8.01 (5.45)	<0.001	
P^1^	0.102	0.104	0.786			

^1^ *p*-value for group comparison ^2^ *p*-value for time comparison ^3^ Repeated measures ANOVA. Effects reported include differences between the groups in the degree of change over the follow-up period. At 24 and 80 h, LS was not detected in plasma samples.

**Table 3:** Changes over the follow-up period in hemodynamic parameters by group.

		Baseline	20 min after CPB	End of Surgery	ICU	Change to ICU		
	Dose	Mean (SD)	Mean (SD)	Mean (SD)	Mean (SD)	Mean (SD)	P^2^	P^3^
HR (beats/min)	6 μg/kg	76.9 (20.7)	86.7 (7.3)	85.1 (8.6)	83.1 (11.9)	6.3 (21.1)	0.251	0.645
	12 μg/kg	69.2 (11.7)	82.1 (10.9)	82.4 (10.8)	80.7 (9.9)	11.5 (15.2)	0.071	
	P1	0.222	0.193	0.452	0.543			
SAP (mm/Hg)	6 μg/kg	110.8 (18.9)	108.1 (13.7)	105.5 (12.9)	116.2 (17.1)	5.4 (24.1)	0.115	0.141
	12 μg/kg	119.9 (20.1)	106.7 (12.1)	103.8 (14.3)	107 (9.1)	−12.9 (20.7)	0.087	
	P1	0.214	0.758	0.73	0.077			
DAP (mmHg)	6 μg/kg	64.9 (11.2)	55.7 (7.9)	59.7 (9)	60.9 (11.1)	−3.9 (9.7)	0.056	0.372
	12 μg/kg	64.8 (15.4)	62.5 (10.2)	59.2 (9.9)	61.3 (8.3)	−3.5 (14.1)	0.566	
	P1	0.989	0.053	0.878	0.912			
MAP (mmHg)	6 μg/kg	82.9 (12.6)	71.9 (8.2)	72.9 (9.3)	78.6 (12.3)	−4.3 (14.6)	0.038	0.509
	12 μg/kg	85.3 (15.8)	69.2 (18.7)	72.9 (9.7)	72.4 (10)	−12.9 (18.6)	0.026	
	P1	0.658	0.608	>0.999	0.142			
CVP (mmHg)	6 μg/kg	15.9 (3.7)	15.7 (2.8)	16.1 (3)	13.7 (2.7)	−2.2 (3.5)	0.042	0.903
	12 μg/kg	15.1 (3.9)	14.7 (3.6)	14.8 (3.4)	13.1 (2.5)	−1.9 (4)	0.138	
	P1	0.538	0.403	0.29	0.532			
SPAP (mmHg)	6 μg/kg	53.1 (7.2)	44.1 (7.3)	44.2 (7)	44.5 (6.9)	−8.5 (10.4)	0.021	0.015
	12 μg/kg	58.9 (12.1)	43.1 (10.3)	42.6 (12.4)	38.4 (9.2)	−20.5 (10.7)	<0.001	
	P1	0.116	0.762	0.668	0.049			
DPAP (mmHg)	6 μg/kg	29.5 (5.4)	25.1 (6.7)	25.8 (4.9)	23.1 (4.5)	−6.5 (6.4)	0.004	0.814
	12 μg/kg	30.1 (7.4)	24.3 (8.1)	24.7 (7.3)	21.8 (6.4)	−8.3 (5.5)	<0.001	
	P1	0.824	0.771	0.643	0.535			
MPAP (mmHg)	6 μg/kg	40 (5)	31.9 (6.5)	33.7 (5.3)	31 (5.5)	−9 (7.8)	<0.001	0.046
	12 μg/kg	42.7 (8.5)	31.7 (8.3)	31.6 (8.2)	27.8 (7.2)	−14.9 (6.6)	<0.001	
	P1	0.291	0.961	0.405	0.182			
MPAP/MAP	6 μg/kg	0.47 (0.09)	0.44 (0.1)	0.46 (0.09)	0.39 (0.08)	−0.08 (0.13)	0.008	0.048
	12 μg/kg	0.52 (0.15)	0.43 (0.13)	0.43 (0.1)	0.34 (0.09)	−0.17 (0.10)	<0.001	
	P1	0.361	0.681	0.350	0.157			
PCWP (mmHg)	6 μg/kg	24.6 (3.5)	23.7 (4.5)	22.9 (4.6)	20.1 (3.4)	−4.5 (3.4)	0.001	0.523
	12 μg/kg	25.2 (4.9)	22.4 (4.7)	21.4 (4.2)	18.4 (2.9)	−6.8 (4.7)	<0.001	
	P1	0.701	0.434	0.347	0.165			
CO (L/min)	6 μg/kg	3.54 (0.68)	4.26 (0.96)	4.27 (0.71)	4.07 (0.72)	0.53 (0.77)	0.022	0.833
	12 μg/kg	4.14 (0.94)	5.03 (0.78)	4.95 (0.72)	4.61 (0.78)	0.47 (0.91)	0.003	
	P1	0.055	0.023	0.015	0.061			
CI (L/min/m^2^)	6 μg/kg	1.96 (0.37)	2.33 (0.49)	2.36 (0.43)	2.24 (0.46)	0.28 (0.41)	0.020	0.742
	12 μg/kg	2.18 (0.51)	2.70 (0.37)	2.61 (0.35)	2.38 (0.39)	0.2 (0.51)	0.001	
	P1	0.201	0.045	0.093	0.386			
SV (mL)	6 μg/kg	49.5 (17.6)	49.2 (11.4)	49.8 (13)	49.6 (10.9)	0.1 (15.6)	0.998	0.709
	12 μg/kg	59.9 (15.6)	62.7 (11.7)	60.7 (10.9)	58 (11.3)	−1.9 (15.4)	0.524	
	P1	0.099	0.003	0.019	0.048			
SVI (mL/m^2^)	6 μg/kg	26.5 (8.8)	27.8 (6.8)	34.9 (21.8)	27.5 (7)	0.9 (8.9)	0.262	0.255
	12 μg/kg	31.7 (8.6)	33.3 (7.5)	32.2 (6.8)	30.7 (6.3)	−1 (8)	0.507	
	P1	0.113	0.042	0.652	0.192			
SVR (dyn*sec/cm^5^)	6 μg/kg	1520.3 (359.8)	1104.1 (301.3)	1074 (249.4)	1347 (423.3)	−173.3 (378.2)	<0.001	0.768
	12 μg/kg	1389.9 (299.7)	982.5 (260.2)	956.4 (198.3)	1122.7 (314.6)	−267.2 (323.3)	<0.001	
	P1	0.29	0.247	0.164	0.111			
SVRI (dyn*sec/cm^5^*m^2^)	6 μg/kg	2804 (664.4)	2112.5 (750.3)	1994.1 (578.1)	2460.1 (813.9)	−343.9 (703.2)	<0.001	0.778
	12 μg/kg	2635.6 (625.5)	1830.2 (372.9)	1796.1 (318.3)	2074.5 (456.8)	−561.1 (643.3)	<0.001	
	P1	0.481	0.203	0.255	0.121			
PVR (dyn*sec/cm^5^)	6 μg/kg	355.4 (100.8)	159.6 (117)	172.5 (73.8)	225.8 (134.3)	−129.6 (179.3)	<0.001	0.654
	12 μg/kg	331.5 (146.7)	147.9 (86.1)	163.6 (88.4)	168.8 (91.1)	−162.7 (129)	<0.001	
	P1	0.608	0.757	0.766	0.185			
PVRI (dyn*sec/cm^5^*m^2^)	6 μg/kg	650 (166.7)	239.2 (195)	315.5 (138.8)	409 (239.2)	−241 (321.9)	<0.001	0.560
	12 μg/kg	619.5 (249.6)	261.8 (194.5)	316.1 (187.3)	321.9 (183.5)	−297.7 (222.4)	<0.001	
	P^1^	0.697	0.753	0.992	0.273			

^1^ *p*-value for group comparison ^2^ *p*-value for time comparison ^3^ Repeated measures ANOVA. Effects reported include differences between the groups in the degree of change over the follow-up period.

**Table 4:** LVEF and RV change over the follow-up period by group.

		Baseline	At the End of Surgery	ICU	Change Until ICU		
	Dose	Mean (SD)	Mean (SD)	Mean (SD)	Mean (SD)	P^2^	P^3^
LVEF (%)	6 mcg/kg	46 (7.6)	47.3 (6.8)	47.3 (7.5)	1.3 (5.2)	0.584	0.881
	12 mcg/kg	49.3 (7.3)	50.3 (4.4)	49.7 (7.7)	0.3 (7.4)	0.703	
	P1	0.231	0.162	0.407			
TAPSE (mm)	6 mcg/kg	15.7 (2.8)	15.7 (2.8)	16.1 (2.7)	0.4 (2.6)	0.264	0.042
	12 mcg/kg	14.5 (1.8)	16.1 (1.4)	16.2 (1.4)	1.7 (0.9)	0.008	
	P^1^	0.198	0.626	0.866			

^1^ *p*-value for group comparison ^2^ *p*-value for time comparison ^3^ Repeated measures ANOVA. Effects reported include differences between the groups in the degree of change over the follow-up period.

## Data Availability

Data is contained within the article and supplementary material.

## Supplementary Materials

The following supporting information can be downloaded at: https://www.mdpi.com/article/10.3390/ph16060815/s1, Table S1: Changes over the follow-up period in LS according to CPB duration. Table S2: SPAP, DPAP, MPAP, and MPAP/MAP changes over the follow-up period, according to LS values at 20 min after CPB. Table S3: LVEF and TAPSE change over the follow-up period, according to LS values at 20 min after CPB. Table S4: ICU and hospitalization duration according to LS values at 20 min after CPB. Figure S1: LC-MS/MS methodology for the determination of LS and its metabolite, OR-1896, using warfarin as internal standard (IS). An indicative MRM chromatogram depicting LS, OR-1896, and IS in concentrations of 0.5 ng/mL, 5 ng/mL, and 1 ng/mL, respectively.

## Appendix A

Data for accuracy and precision of the developed LC-MS method for the quantification of LS and OR-1896 in human plasma. The nominal concentration of analytes in low QC: 0.2 ng/mL LS, 2 ng/mL OR-1896; in middle QC: 20 ng/mL LS, 20 ng/mL OR-1896 and in high QC: 40 ng/mL LS, 40 ng/mL OR-1896. Each run included (*n* = 2 of samples).

**Table A1:** Results from three different analytical runs for intra-assay accuracy determination for the quantification of LS and OR-1896 in human plasma.

Within Run Accuracy	Low QC		Middle QC		High QC	
	Mean Calculated Concentration (ng/mL)	Accuracy %	Mean Calculated Concentration (ng/mL)	Accuracy %	Mean Calculated Concentration (ng/mL)	Accuracy %
1st _(n = 4)_						
LS	0.218	110	20.0	99.8	37.5	93.7
OR-1896	2.078	104	21.0	105	39.9	100
2nd _(n = 2)_						
LS	0.210	105	18.8	94.1	36.2	90.4
OR-1896	1.915	95.7	17.9	89.6	35.2	87.9
3rd _(n = 2)_						
LS	0.198	98.8	19.1	95.2	40.1	100
OR-1896	1.945	97.2	18.6	92.6	40.4	101

**Table A2:** Overall results for accuracy and precision between three analytical runs for the quantification of LS and OR-1896 in human plasma.

Between Runs	Low QC			Middle QC			High QC		
	Mean Calculated Concentration (ng/mL)	Accuracy %	Precision %	Mean Calculated Concentration (ng/mL)	Accuracy %	Precision %	Mean Calculated Concentration (ng/mL)	Accuracy %	Precision %
LS	0.209	105	4.8	19.3	96.4	3.2	37.9	94.7	5.2
OR-1896	1.979	99.0	4.4	19.2	95.7	8.5	38.5	96.3	7.5
