# Supplementary material for: Monitoring of Levosimendan Administration in Patients with Pulmonary Hypertension Undergoing Cardiac Surgery and Effect of Two Different Dosing Schemes on Hemodynamic and Echocardiographic Parameters
Source: Pharmaceuticals (Basel). 2023 May 30;16(6):815. doi: 10.3390/ph16060815 (PMC10305589; doi:10.3390/ph16060815)

pharmaceuticals

Article

Monitoring of levosimendan administration in patients with pulmonary hypertension undergoing cardiac surgery and effect of two different dosing schemes on hemodynamic and echocardiographic parameters

Panagiotis Ftikos[1], Areti Falara[1], Panagiota Rellia[1], Evangelos Leontiadis[2], George Samanidis[3], Natalia Kamperi[4], Artemios Piperakis[4], Constantin Tamvakopoulos[4], Theofani Antoniou[1] and Kassiani Theodoraki[5]

[1]Department of Anesthesiology, Onassis Cardiac Surgery Center, Athens, Greece
[2]Department of Cardiology, Onassis Cardiac Surgery Center, Athens, Greece
[3]Department of Cardiac Surgery, Onassis Cardiac Surgery Center, Athens, Greece
[4]Center of Clinical Research, Experimental Surgery and Translational Research, Division of Pharmacology-Pharmacotechnology, Biomedical Research Foundation, Academy of Athens, Athens, Greece
[5]Department of Anesthesiology, Aretaieion University Hospital, National and Kapodistrian University of Athens, Greece

* Correspondence: Kassiani Theodoraki MD, PhD, DESA; Department of Anesthesiology; Aretaieion University Hospital; National and Kapodistrian University of Athens, Greece; Email: ktheod@med.uoa.gr; Orcid ID : 0000-0003-4890-4642

Table S1. Changes over the follow-up period in LS according to CPB duration.

	LS					
	20 min after CPB	6 hours	12 hours	Change to 12 hours		
Group	Mean (SD) (ng/ml)	Mean (SD) (ng/ml)	Mean (SD) (ng/ml)	Mean (SD) (ng/ml)	P[2]	P[3]
CPB duration<166 min	8.69(4.78)	3.46(2.38)	.19(.20)	-8.50 (4.68)	<0.001	0.001
CPB duration>166 min	2.87(1.81)	1.06(.65)	.18(.22)	-2.68 (1.83)	0.127	
P[1]	0.001	0.004	0.931			

[1]p-value for group comparison [2] p-value for time comparison [3]Repeated measures ANOVA. Effects reported include differences between the groups in the degree of change over the follow-up period. CPB duration was dichotomized according to upper tertile that was equal to 166 min, into two groups. LS values during follow up according to CPB duration are shown in supplementary table 1. Significantly greater mean values of LS were found at 20 min after CPB and at 6 hours after CPB for those whose CPB duration was less than 166 minutes. At 12 hours there was no significance difference in LS levels according to CPB duration. LS levels were significantly reduced in both groups over time but the reduction was greater in those with CPB duration<166 min.

At 24 and 80 hours, LS was not detected in plasma samples.

Table S2. SPAP, DPAP, MPAP and MPAP/MAP changes over the follow-up period, according to LS values at 20 min after CPB.

	LS 20 min after CPB	Baseline	20 min after CPB	End surgery	ICU	Change to ICU		
		Mean (SD)	Mean (SD)	Mean (SD)	Mean (SD)	Mean (SD)	P[2]	P[3]
SPAP (mmHg)	<6.7 ng/ml	55.9 (12.4)	45.2 (10.8)	43 (11)	40.6 (9.2)	-15.3 (12)	0.001	0.509
	>6.7 ng/ml	56.1 (7.8)	42.1 (6.3)	43.8 (9.2)	42.3 (8.2)	-13.8 (12.5)	<0.001	
	P1	0.944	0.339	0.830	0.590			
DPAP (mmHg)	<6.7 ng/ml	29.5 (7.1)	25.1 (8.6)	25.1 (7)	22.5 (6.4)	-7 (6.9)	0.003	0.911
	>6.7 ng/ml	30.1 (5.9)	24.3 (6.1)	25.5 (5.3)	22.3 (4.6)	-7.7 (5.1)	0.001	
	P1	0.824	0.771	0.862	0.922			
MPAP (mmHg)	<6.7 ng/ml	41.5 (9)	32.7 (8.7)	32.2 (7.2)	29 (7.2)	-12.5 (8.8)	<0.001	0.632
	>6.7 ng/ml	41.2 (4.4)	30.9 (5.8)	33.1 (6.7)	29.8 (6)	-11.4 (6.7)	<0.001	
	P1	0.899	0.496	0.717	0.742			
MPAP/MAP	<6.7 ng/ml	0.48 (0.13)	0.46 (0.12)	0.46 (0.1)	0.39 (0.11)	-0.09 (0.12)	0.008	0.284
	>6.7 ng/ml	0.51 (0.12)	0.41 (0.1)	0.43 (0.09)	0.37 (0.07)	-0.14 (0.11)	<0.001	
	P[1]	0.479	0.307	0.459	0.470			

[1]p-value for group comparison [2] p-value for time comparison [3]Repeated measures ANOVA. Effects reported include differences between the groups in the degree of change over the follow-up period.

Table S3. LVEF and TAPSE changes over follow-up period, according to LS values at 20min after CPB.

	LS 20min after CPB	Baseline	at the end of surgery	ICU	Change until ICU		
		Mean (SD)	Mean (SD)	Mean (SD)	Mean (SD)	P[2]	P[3]
LVEF (%)	<6.7 ng/ml	49.7 (6.7)	48.7 (4.8)	49.3 (6.2)	-0.3 (5.8)	0.640	0.101
	>6.7 ng/ml	45.7 (8)	49 (6.9)	47.7 (8.8)	2 (6.8)	0.023	
	P1	0.148	0.879	0.555			
TAPSE (mm)	<6.7 ng/ml	16.1 (2.4)	16.2 (2.7)	16.3 (2.7)	0.1 (2.3)	0.939	0.015
	>6.7 ng/ml	14.1 (2)	15.5 (1.5)	16 (1.4)	1.9 (1.2)	0.001	
	P[1]	0.070	0.415	0.736			

[1]p-value for group comparison [2] p-value for time comparison [3]Repeated measures ANOVA. Effects reported include differences between the groups in the degree of change over the follow-up period.

Table S4. ICU and hospitalization duration according to LS values at 20 min after CPB.

	LS 20min after CPB		
	<6.7 ng/ml	>6.7 ng/ml	P
ICU length of stay (hours), mean (SD)	108 (52.2)	130.9 (265.3)	0.753+
Duration of hospitalization (days), median (IQR)	8.5 (7 — 12)	9 (7 — 10)	>0.999++

+Student's t-test; ++Mann-Whitney test.

Figure S1: LC-MS/MS methodology for the determination of LS and its metabolite, OR-1896, using warfarin as internal standard (IS). An indicative MRM chromatogram depicting LS, OR-1896 and IS in concentrations of 0.5 ng/mL, 5 ng/mL and 1 ng/mL, respectively.

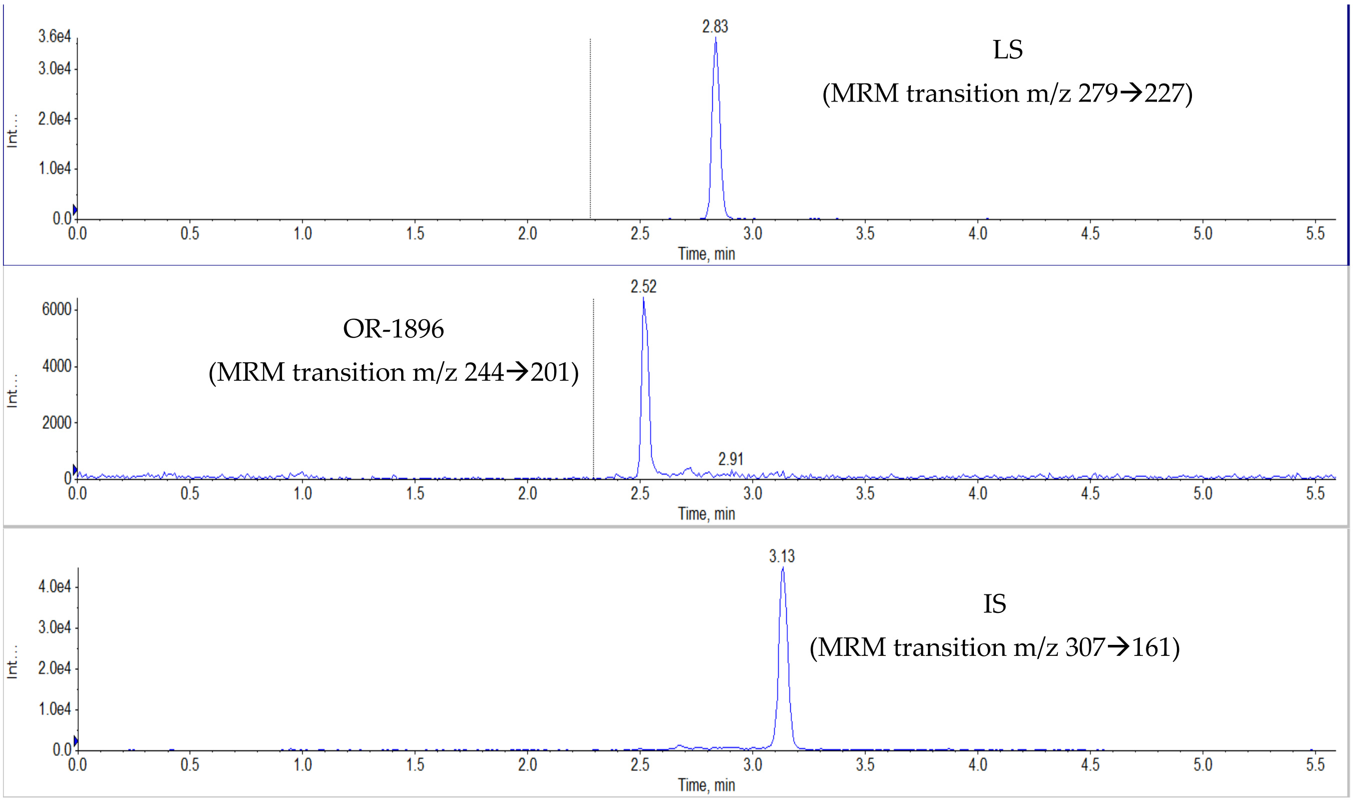

Supplement: Supplementary file 1 [file pharmaceuticals-16-00815-s001.zip › pharmaceuticals-2306287-supplementary.pdf]
